# Building Resilience and Attachment in Vulnerable Adolescents: The Feasibility of a Virtually Delivered Group Intervention for Adolescents with Suicidal Ideation and Their Caregivers

**DOI:** 10.1089/tmr.2023.0056

**Published:** 2024-02-13

**Authors:** Nicole Sheridan, Allison Kennedy, Clare Gray, Leigh Dunn, Jayme Stewart, Hannah Elliott, Kendra Carnrite, Ademola Adeponle, Paula Cloutier

**Affiliations:** ^1^ Psychiatry and Mental Health Research, Children's Hospital of Eastern Ontario Research Institute, Ottawa, Ontario, Canada.; ^2^ Mental Health, Children's Hospital of Eastern Ontario, Ottawa, Ontario, Canada.; ^3^ Department of Psychiatry, Faculty of Medicine, University of Ottawa, Ottawa, Ontario, Canada.

**Keywords:** adolescence, caregivers, group therapy, suicidal ideation, telehealth

## Abstract

**Background:**

The COVID-19 pandemic required many interventions to be conducted virtually. Building Resilience and Attachment in Vulnerable Adolescents (BRAVA) is a group intervention designed for adolescents and their caregivers to reduce adolescent suicidal ideation (SI).

**Objective:**

We aimed to adapt BRAVA for virtual delivery and evaluate its acceptability and feasibility.

**Methods:**

We conducted an 8-week pre–post trial between October and December 2020. Six adolescents and six primary caregivers were recruited from a pediatric hospital in Ontario, Canada. Families completed a virtual intake and exit assessment together and 6 weekly BRAVA group sessions separately. Satisfaction feedback was collected after each group session and during their exit, and clinical outcomes were collected at intake and exit. Weekly team meetings were conducted to gather clinician feedback.

**Results:**

The study uptake rate was 42.9% of eligible participants. There were no dropouts. Adolescent and caregiver attendance rates for group sessions were high (median = 6). Most youth (83.4%) and caregivers (66.7%) reported that the virtual process worked well. All caregivers (100%) agreed they would participate in a virtual group session again, compared with youth (50%). Providers approved of the virtual adaptation but identified potential improvements (e.g., manual content, safety procedures). Adolescent SI decreased significantly post-treatment (*M*_pre_ = 50.7, *M*_post_ = 29.7, *p* = 0.002).

**Conclusions:**

Virtual delivery of BRAVA is acceptable and feasible and may help reduce SI in adolescents. Uptake, retention, and satisfaction were high for adolescents and caregivers. Feedback collected will improve BRAVA for future evaluations, including a randomized controlled trial.

## Introduction

### Telehealth and suicidal risk

The COVID-19 pandemic required health care providers to rapidly shift to providing telehealth and virtual care options. Pediatric telemedicine services have been suggested as comparable with or better than in-person services.^1^ However, mental health (MH) telehealth services come with unique challenges for youth presenting with suicidal ideation (SI), whereby effective connection (interpersonally and technically) is essential for safety.^2–4^ Evaluation of telehealth treatments for adolescents with SI is lacking, despite increasing rates of SI during the pandemic.^5–8^

### Virtual group interventions

At the beginning of the pandemic, group interventions lagged behind other treatment modalities in the pivot toward telemedicine as not all therapies translated as smoothly as others, possibly due to administrative coordination and clinical content adaptation needs.^9^ One caregiver survey study reported a moderately high acceptability rate for virtual groups where lectures, group discussions, and using chat functions were noted as the most acceptable features.^10^

Among group-based interventions, dialectical behavioral therapy (DBT) has demonstrated the most overall efficacy in reducing adolescent SI,^11^ but research in its virtual adaptability is limited. A review of the existing literature examining online DBT suggests that this approach is acceptable and feasible; however, adaptations may be required to address concerns related to privacy, technology, and engagement.^12,13^ Notably, only one study evaluated a virtually adapted DBT with pediatric clients, based on qualitative clinician feedback.^14^

This study identified interactive features to enhance client engagement and group cohesion, a facilitator for technology issues, and follow-up procedures for early session departure as important components of a virtual adaptation. In another qualitative study examining online DBT, Bock et al. described how adolescent participants benefitted from the intervention, but that technology issues interfered with their therapeutic experience.^15^ Both studies discussed above did not include caregivers, and to date, we are unaware of any studies evaluating youth and caregiver satisfaction within virtual group interventions for reducing adolescent SI.

### Building Resilience and Attachment in Vulnerable Adolescents overview

Building Resilience and Attachment in Vulnerable Adolescents (BRAVA) is a brief group intervention for adolescents with mild-to-moderate SI and their caregivers.^16^ The primary treatment goals are to reduce SI in adolescents and increase family connection. BRAVA consists of six 90-min modules that are delivered in a group setting on a weekly basis. The modules include components of DBT, attachment-based family therapy, cognitive behavioral therapy, collaborative problem solving, and psychoeducation. Adolescents and their caregivers attend separate groups. BRAVA modules were adapted for virtual delivery to continue its evaluation of reducing adolescent SI during the pandemic.

## Objective and Hypothesis

The goals of this study were to (1) adapt BRAVA for virtual delivery, (2) evaluate the feasibility and acceptability of this adaptation, and (3) identify the improvements needed to optimize virtual delivery in planning for a randomized controlled trial. We hypothesized that BRAVA's virtual delivery would be feasible from a procedural standpoint and acceptable to participants and facilitators.

## Methods

Ethics approval was granted from the CHEO Research Ethics Board (19/22E) and reported based on Transparent Reporting of Evaluations with Nonrandomized Designs guidelines. All participants provided informed consent.

### Study design

This study was an 8-week nonrandomized pre–post trial conducted from September to December 2020.

### Participants

Participants were recruited by clinicians in the emergency department, an MH outpatient clinic or an MH centralized intake service at a pediatric hospital in Ontario, Canada. Participants met inclusion criteria if they had mild-to-moderate SI, rated as a score of 1 (no immediate intent and the ability to safety plan) on the HEADS-ED screening tool,^17,18^ were between 13 and 17 years old, and had access to an electronic device, with microphone and camera.

Exclusion criteria included SI with a plan or suicidal gesture (2 on HEADS-ED Suicide item); psychosis, schizophrenia, developmental disabilities, conduct disorder or major substance abuse; current MH services involvement at least weekly; under the care of child protection services (as the BRAVA intervention requires caregiver participation); and expressed difficulty with reading and/or writing.

### Procedures

All study consent forms and questionnaires were administered through Research Electronic Data Capture,^19^ except for the Suicidal Ideation Questionnaire-Junior (SIQ-JR). Consent forms were signed by both adolescents and their primary parent/caregiver. Intake and exit assessments, as well as group sessions were conducted through a secured Zoom account that met Canadian health care privacy standards. All assessments were completed by master's level research assistants (RAs) trained to conduct suicide risk assessments and engage in safety planning in a virtual context; a psychologist or psychiatrist was on call for consult during the assessment and before study enrollment.

#### Intake assessment

Consenting adolescents and parent/caregiver(s) were e-mailed the intake assessment information, including a web link to complete an electronic consent form and questionnaires before the assessment. Research staff scored and reviewed the results before the assessment. The RA began the session by confirming the family's current location/address and contact information and the caregiver was required to be in the home entirely during the assessment (safety protocol).

The RA reviewed study details with the family and then met privately with the adolescent to administer further questionnaires, conduct a risk assessment, and develop a safety plan. With adolescent consent, this information was shared with the caregiver. Caregivers were routinely provided standard safety information pertaining to supporting a child with SI (e.g., monitor their child, secure potentially lethal means when necessary). All eligible families were enrolled to participate in the BRAVA intervention.

#### BRAVA groups

Youth and caregivers each attended six weekly 90-min groups, which were conducted on separate days to avoid internet bandwidth issues and device availability for participants in the same household. Adaptations were made to the intervention to increase engagement within the virtual setting (Table 1).

**Table 1.** Adaptations Made for Virtual Delivery of Building Resilience and Attachment in Vulnerable Adolescents

Component added for virtual delivery	Description
Before group	
Group reminders	Participants received a weekly e-mail with a reminder of the group date/time, the Zoom link, and the handouts to use during that week's group
Emergency contact list	A weekly emergency contact list was sent to all research staff with each family's information in the event of safety concerns and included the youth and parent/caregiver(s) names, contact information, and home address
During group	
Research team	Each group session included three study facilitators, a senior clinician (psychiatrist or psychologist), child and youth counsellor or a master's level researcher) and a research assistant to coordinate technology needs (e.g., PowerPoint, [Microsoft Corporation, 2020]), monitor the chat for questions, and help participants troubleshoot issues
PowerPoint slides	PowerPoint presentations were created for all modules to complement the facilitator manual. Slides were colorful and inviting and included didactic content, fillable tables (instead of using flip charts or whiteboards that were used previously for in-person groups), brief activities to allow for reflection and/or practice of coping strategies, and brief videos relating to the module topic
Zoom etiquette	Each session began with a “Zoom Etiquette” slide outlining the rules of participating in a virtual group, including adding their name and pronouns, keeping cameras on, prohibiting taking pictures or videos of the screen, to ensure they are in a private space, to keep their microphone muted when not speaking and raise hand if they have a question or comment, minimize distractions and limit background noise, and instructions for how to view participants and PowerPoints at the same time
Handouts	Any handouts that were initially created for exercises as part of the modules were adapted to fillable PDFs that were e-mailed to participants before each group. Participants could choose to print them or complete them electronically
Module activities requiring specific objects	Activities involving tangible objects were also adapted to be based on what youth might have in their room. For example, the adolescent module *Surviving a Crisis* previously included a short activity of creating a crisis box with coping cards and this activity was removed from the virtual adaptation all participants might not have the necessary materials
After group	
Satisfaction survey	At the end of each module, a link to a brief satisfaction survey was e-mailed to all participants and shared in the Zoom chat. Participants were asked to complete it before signing off

#### Exit assessments

**FIG. 1.**
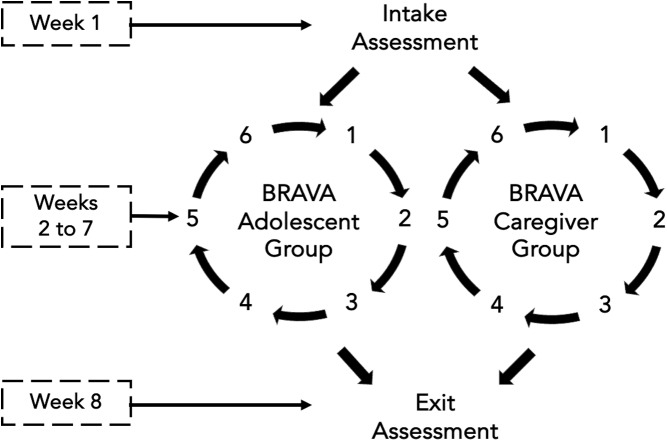
BRAVA intervention flowchart. BRAVA, Building Resilience and Attachment in Vulnerable Adolescents.

After module six, exit assessment information, including questionnaire link, was e-mailed to participants. Exit assessment procedures were identical to the intake assessment but included questions about their experience with the virtual format. Adolescent participants received a gift card after the assessment. See Figure 1 for study flowchart.

### Measures

#### Demographics

Adolescent and caregiver demographic data (i.e., age, gender, ethnicity, and current use of psychotropic medications for adolescents; age, relation to child, annual household income, whether the caregiver had an MH diagnosis) were collected using a self-report survey developed by the study team. Ethnicity variables were based on the Government of Canada's 2016 Census Profile.^20^

#### Clinical questionnaires

##### HEADS-ED

The HEADS-ED^17,18^ is a seven-item MH screening tool for children and youth that assesses level of need and functioning across seven domains. The items are scored on a scale of 0 (*no action needed*), 1 (*needs action but not immediate/moderate functional impairment*), or 2 (*needs immediate action/severe functional impairment*). Items can stand on their own or be tallied into a total score. This validated tool was used to assess for study inclusion criteria for suicidality and to describe the level of functioning/need in study participants.

##### Suicidal Ideation Questionnaire-Junior

The SIQ-JR^21^ is a 15-item version of the SIQ for adolescents aged 15–18 years. The SIQ-JR assesses SI within the last month on a scale of 0–6 (0 = *I never had this thought*; 6 = *almost every day*). A score of 31 or greater is above the clinical cutoff. The SIQ-JR has a 3-week test–retest reliability of 0.89.^22^

##### Revised Child Anxiety and Depression Scale

The Revised Child Anxiety and Depression Scale (RCADS) is a 47-item questionnaire that screens for adolescent depression and anxiety. Items are scored from 0 to 3 (0 = *never*, 3 = *always*). *T*-scores >80 are considered above the clinical cutoff. Internal consistency alphas range from 0.78 to 0.88, and 1-week test–retest reliabilities range from 0.65 to 0.8.^24^

#### Outcome measures

##### Treatment module satisfaction

The study team developed a 12-item questionnaire that was completed by participants after each group. Satisfaction was rated from 0 to 5 (0 = *Not at all useful*; 5 = *Very useful*) for each topic and handout of the BRAVA module, the comprehension and relevance of the topics, and information about their technology experience during the group. A final open-ended question allowed participants to provide additional feedback on the session.

##### Virtual experience satisfaction

The study team developed a 30-item questionnaire related to the participant's experience with virtual assessments and groups. These questions were rated from 0 to 5 (1 = *Strongly disagree*; 5 = *Strongly agree*). Additional questions asked whether specific virtual features should be used more, about the same, or less, and five open-ended questions about their experiences during the group.

### Module fidelity

All group sessions were audio recorded to assess for treatment fidelity. Two RAs reviewed audio recordings to ensure all group components were delivered based on the intervention manual.

### Identifying improvements

Weekly meetings were held with facilitators to discuss barriers encountered while delivering BRAVA virtually. Barriers and solutions were tracked and categorized by participant safety, technology, participant engagement, administration, and module content.

### Data analyses

Descriptive statistics were used to analyze the uptake rate, retention rate, participant satisfaction ratings, and clinician adherence to the treatment manual. Pre- and postanalyses on self-report measures (SIQ-JR and RCADS) were done using two-tailed *t*-tests for matched pairs. Qualitative feedback from participants was coded using thematic analysis by two RAs with conflicts resolved by a third coder. All quantitative analyses were completed using SPSS version 27.^25^ All qualitative analyses were completed using Microsoft Excel.^26^

The number of families to recruit for the one cycle was determined based on previous studies of BRAVA where the median group size was five adolescents,^16^ and DBT-A with suicidal adolescents' recommendations for group size that range from four to six families.^27^

## Results

### Participant flow and characteristics

**FIG. 2.**
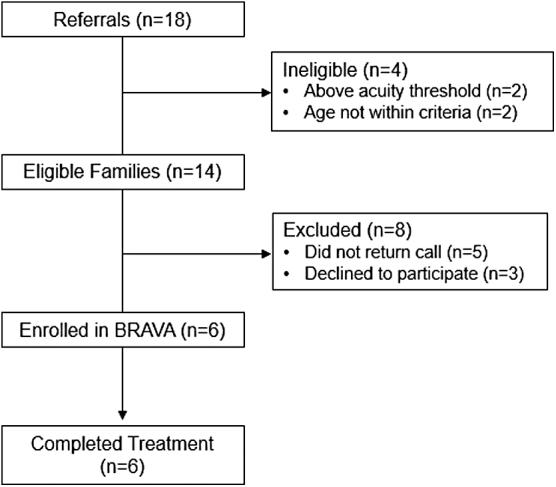
Participant flow.

Study participant flow is shown in Figure 2. Of the eligible families referred (*n* = 14), 42.9% (*n* = 6) were enrolled and retention was complete (*n* = 6, 100%). Of the eight families who were referred to the study but did not participate, four were unable to be reached for consent, and the other four expressed they were not interested in participating in the study.

Adolescent participants had an average age of 15.33 years (standard deviation = 1.03; 95% confidence interval [14.506–16.154]) and were mostly female (*n* = 4, 66.7%), of ethnically diverse backgrounds including European, Latin/Central South American, North American Aboriginal, African, and Caucasian. Half were prescribed psychotropic medications before beginning the study (*n* = 3, 50%).

Clinical characteristics are summarized in Table 2. The average HEADS-ED score indicates a moderate level of need and functioning. SIQ-JR and RCADS scores fell from above their clinical cutoffs at pretreatment to below cutoffs post-treatment. The change in SIQ-JR scores was considered statistically significant (*p* = 0.002).

**Table 2.** Clinical Characteristics

	Pretreatment, mean (SD)	Post-treatment, mean (SD)	***t***	***p***	95% CI
HEADS-ED	5.17 (1.33)	N/A			
SIQ-JR	50.67 (16.7)	29.7 (20.4)	5.7	0.002	11.52–30.5
RCADS (T-score)	67 (10.28)	60.5 (16.08)	1.02	0.353	-9.82–22.82

95% CI, 95% confidence interval of the difference; N/A, not applicable; RCADS, Revised Child Anxiety and Depression Scale; SD, standard deviation; SIQ-JR, Suicidal Ideation Questionnaire-Junior.

### Intervention characteristics

Across the six modules, the adolescent group averaged 5.3 participants, whereas all caregiver participants attended all groups. Two adolescent participants missed two groups each, whereas the rest attended all six sessions. Treatment manual compliance was 100%, indicating no module components were missed.

### Participant satisfaction

#### Virtual BRAVA satisfaction

**FIG. 3.**
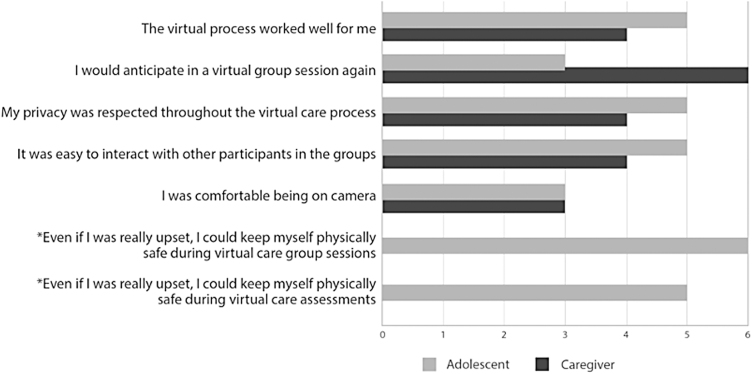
Participants who agreed to the satisfaction statements. *Indicates questions only asked to youth participants and not caregivers.

Figure 3 portrays the satisfaction of the intervention overall. Most participants agreed that the virtual process worked well for them. All caregivers and half of the adolescents reported that they would participate in a virtual group session again. All adolescents reported being able to manage their safety during group sessions, even when upset.

**FIG. 4.**
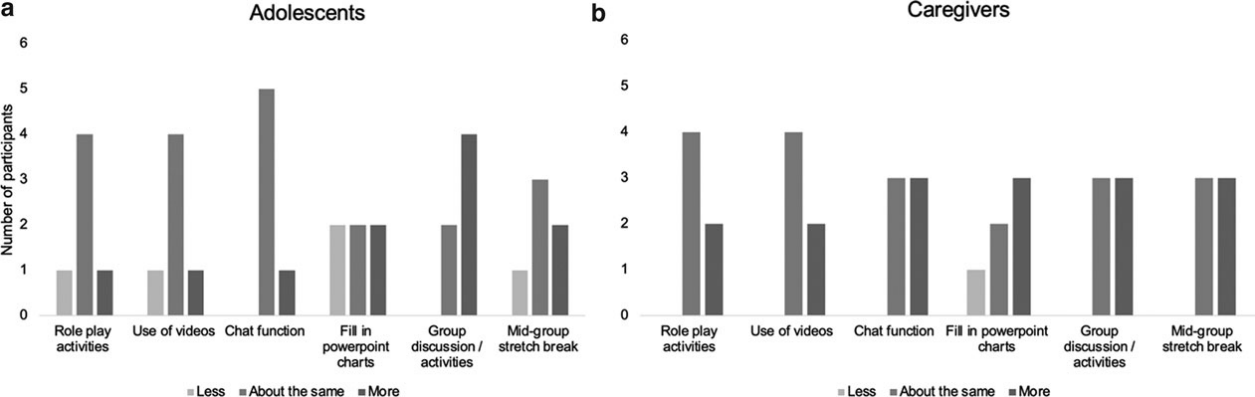
Adolescent **(a)** and caregiver **(b)** preferences for characteristics of delivering therapeutic content virtually.

Participants were generally satisfied with the methods used to deliver therapeutic content (Fig. 4). Many adolescents and caregivers wanted more opportunities for discussion but were satisfied with the number of role play activities and videos.

#### Qualitative themes

Three themes emerged from the youth and caregiver responses related to experiences participating virtually in the BRAVA intervention: sense of peer support, positive educational experience, and recommendations for improvements.

##### Sense of peer support

Participants appreciated the feeling of connection and peer support among group members. Most caregivers expressed that the best thing about coming to the group was knowing they were not alone. The adolescents echoed this normalization such that they felt less alone when they realized other teens go through similar experiences.

I found it very nice to have people who knew what I was talking about and understood my problems/situations.—BRAVA youth

Feeling less alone, getting support, [and] sharing experiences.—BRAVA caregiver

##### Positive educational experience

The second major theme that emerged was that BRAVA was a positive educational experience in which participants learned practical skills. Caregivers reported that psychoeducation was the most helpful therapeutic technique, particularly skills related to validation, being present and connected with their teens, and information regarding teen behaviors, and crisis resources to access when their teens experience suicidal thoughts. The youth also identified useful practical skills including education about sleep hygiene and how to better cope with stress.

This group has led me to make changes necessary to better help my child. Picking apart sections each week to focus in on and practice was very beneficial to me. Learning pace was excellent.—BRAVA caregiver

The most useful thing I learnt was how to better cope with stress and my feelings.—BRAVA youth

##### Recommended improvements to BRAVA

Lastly, both youth and caregivers identified areas for improvement to enhance the content and virtual delivery of BRAVA. The caregivers recommended improvements to group timing and content. One caregiver reported the group was too long and one reported it was too short as they wanted more participant discussion. Other caregivers also wanted more time to share their experiences and learn from other caregivers. One caregiver identified additional topics of potential value and interest, including dealing with anger/physical outbursts, the impact of adolescent MH issues on the family, and parenting multiple children.

One youth reported experiencing little benefit from the topic of online safety as the family already discussed this topic at home. Potential topics of interest identified by youth were how to deal with lack of sleep, trauma, and learning how to be happy and more confident with themselves.

More interactive discussion on the topics amongst the families and what worked/didn't work for each other—BRAVA caregiver

I wish that we could have gone into more in depths about how to be happy with yourself or to be more confident.—BRAVA youth

### Feedback from facilitators

Through study team discussion, group facilitators identified concerns about addressing adolescent safety regarding suicidal risk in a virtual environment. Other identified barriers included participant technology difficulties, maintaining participant engagement, and administrative difficulties (e.g., questionnaire completion).

### Improving the virtual BRAVA intervention

Several improvements to the intervention were made during and after the study was completed (Table 3), including a subsequent randomized controlled trial. Safety, technology, and administrative issues were addressed by obtaining more information (e.g., participant address) or providing more concrete information (e.g., weekly reminder texts, expectations regarding camera use). To increase engagement, we reduced time on didactic content, increased activity and discussion time, and integrated other features (e.g., soothing music).

**Table 3.** Summary of Improvements Made

Category	Barrier encountered	Improvement made
Participant safety	Clinician concern about managing elevated participant SI in a virtual setting	Ask youth to identify location, contact information, and emergency contact each week
Technology	Participant uncertainty regarding navigating a virtual meeting platform	Provide specific instructions of how to use Zoom at the beginning of each group (e.g., how to change their name on the screen)
	Participant reports of missed e-mails with details of the group sessions	Provide weekly texts to participants with Zoom link
	Youth not keeping cameras on	Including expectation of cameras being on at the beginning of group and providing rationale
Participant engagement	Participants' difficulty maintaining attention throughout entire session	1. Decrease time spent delivering didactic content, and increase time spent on activities and group discussions 2. Soothing music during activities 3. Breaking topics down into smaller steps
Administration	Difficulty with questionnaire completion when sending through e-mail	Reminders sent through e-mail on days leading up to assessment, and text message and phone reminders on the day of
Groups	Participants not being in a private space	Setting ground rules during intake session. Weekly reminder through e-mails and at the beginning of each group session
	Participants not being able to access electronic handouts or print	Began printing and mailing out handouts to new participants when they began group
Modules	Difficulty completing modules within allocated timeframe of 90 min	Shortened modules by streamlining didactic components to be more direct and straightforward and/or removing topics that were difficult to complete over Zoom
	Some topics were not as engaging when presented over Zoom (e.g., extensive discussion on technology for teens and online safety based on participant feedback that this topic is discussed extensively already in their lives)	Adapted modules to remove direct didactic teaching on online safety and technology, and discussed these topics within the context of bullying and conflict resolution

SI, suicidal ideation.

## Discussion

BRAVA is a brief group intervention for adolescents with mild-to-moderate SI and their caregivers with encouraging preliminary results for decreasing adolescent SI in a previously conducted pre–post trial.^16^ This study demonstrated that the virtual delivery of BRAVA is feasible and acceptable, and identified potential areas of improvement to be integrated for further evaluations of BRAVA. Based on participant and facilitator feedback, areas of improvement included preparing Zoom-specific resources for families, modifying module content and how it was presented, and communication methods to improve participant engagement.

Study enrollment, retention, and satisfaction were high across both youth and caregivers. Although determining treatment outcome was not identified as a goal for this pilot study, adolescent SI decreased, and fell below clinical cutoffs, post-treatment.

This study meets the need of demonstrating the feasibility of suicide-specific treatments conducted virtually, as outlined in a recent scoping review of telehealth treatments for suicidal clients.^28^ Our results provided additional evidence that the BRAVA intervention is feasible when delivered virtually. All participants were retained for the duration of the intervention that is consistent with previous studies that have demonstrated a greater participation rate for virtual care.^28^ Feedback from our participants was generally positive and consistent with other telehealth studies in adolescent group interventions.^13,15^

Major themes identified through qualitative feedback from adolescents and caregivers revealed that the sense of peer support fostered by sharing and relating to others in the group was beneficial. In addition, participants found virtual BRAVA to be a positive educational experience where they were able to learn new skills from facilitators and group members. Recommendations for improvement included increasing opportunity for discussion and other topics of interest (e.g., increasing adolescent self-confidence). These findings are consistent with previously published qualitative studies on conducting child and adolescent groups virtually, such as challenges with safety management, technology, and participant engagement.^14,15^

Providing more support to navigate the virtual environment (e.g., didactic information, designated facilitator role), opportunities for active participation, and text/e-mail reminders may improve participant engagement and overall experience (e.g., fewer interruptions related to technology issues). These findings are similar to previous evaluations of BRAVA, whereby adolescent participants experienced a decrease in SI after completion of the intervention.^16^ To our knowledge, BRAVA is the first group intervention for suicidal adolescents that has tested its virtual adaptation to ensure that pre- and postoutcomes are consistent with in-person delivery, along with extensive satisfaction questionnaires from adolescents and caregivers to improve the virtual intervention.

With respect to study limitations, although our sample was diverse with respect to ethnicity and youth gender, our sample size was small, was recruited from a single facility, and caregiver participants were primarily maternal and had a high average household income. Therefore, participants had the resources (e.g., technology equipment, reliable internet, a private location in the family home) to participate in a virtual intervention. Families with less resources may encounter more barriers to participating, due to technology availability and privacy. Any conclusions regarding treatment efficacy can be speculative at best given the small sample size and lack of control group.

However, even with a very small sample size, improvements in adolescent SI are consistent with the previous study.^16^ A randomized controlled trial was recently completed to further evaluate the virtual BRAVA intervention,^29^ which included many improvements identified as a result of this pilot study.

## Conclusion

This study indicates that the virtual delivery of BRAVA is feasible and acceptable to adolescents and their caregivers. Conducting BRAVA virtually has the potential to be beneficial for adolescents who experience SI and their caregiver(s). Participant feedback informed changes to the BRAVA intervention for use in a randomized controlled trial to determine treatment effectiveness.

## Data Availability

The data sets generated during and/or analyzed during this study are available from the corresponding author on reasonable request.

## Abbreviations Used

95% CI: 95% confidence interval of the difference
BRAVA: Building Resilience and Attachment in Vulnerable Adolescents
DBT: dialectical behavioral therapy
MH: mental health
RA: research assistants
RCADS: Revised Child Anxiety and Depression Scale
SD: standard deviation
SI: suicidal ideation
SIQ-JR: Suicidal Ideation Questionnaire-Junior
